# Establishment of an optimized orthotopic bladder cancer model in mice

**DOI:** 10.1186/s12894-022-01093-6

**Published:** 2022-09-03

**Authors:** Jinming Cai, Zhiwen Xie, Yilin Yan, Zhengnan Huang, Pengfei Tang, Xiangqian Cao, Zeyi Wang, Chenkai Yang, Mingyue Tan, Fang Zhang, Bing Shen

**Affiliations:** 1 Department of Urology, Shanghai General Hospital, Shanghai Jiaotong University School of Medicine, No. 85 Wujin Road, Hongkou District, Shanghai, 200080 China; 2 Department of Urology, Shanghai General Hospital Affiliated to Nanjing Medical University, Shanghai, 200080 China; 3 Department of Urology, Shuguang Hospital, Shanghai University of Traditional Chinese Medicine, Shanghai, 200021 China

**Keywords:** Bladder cancer, Orthotopic murine model, Microsyringes, MB49

## Abstract

**Background:**

Bladder cancer (BC) is one of the most common malignancies of the genitourinary system. Animal models offer an important tool to explore tumour initiation, progression, and therapeutic mechanisms. Our aim is to construct an optimized orthotopic BC model which is predictable, reproducible, and convenient.

**Methods:**

The optimized orthotopic BC model was constructed in male C57BL/6 mice utilizing microsyringes to inoculate them with a murine BC cell line (MB49). Anesthetised mice were inoculated with an MB49 cell suspension (10 µL) at approximately 5 × 10^6^/mL. The whole process of modelling was observed and monitored every 3 days for 21 days utilizing HE staining and transabdominal ultrasonography (TUS).

**Results:**

In this study, the model showed excellent success rates for tumour formation (96.67%) and metastatic rate (89.66%). Compared to the control group (sham operation), mice in the modelling group had serous cachexia, visible haematuresis and weight loss (all *P* < 0.05). The lungs, liver, ureter and kidneys were found to have tumour metastasis. Moreover, the average survival time (19.73 ± 1.69 d) of modelling mice was significantly shorter than that of the control mice (*P* < 0.05), which remained alive.

**Conclusion:**

Our study established a method using microsyringes to inject murine BC cells into the bladder wall, creating a stable transplantable BC model in mice.

## Introduction

Bladder cancer (BC) is one of the most common urogenital malignancies, which threatens human health for many years. The global incidence of BC ranks tenth, with up to 573,278 cases in 2021 [1]. According to the clinical pathological classification, bladder urothelial carcinoma accounts for 90–95% of BC cases, and the rest are bladder adenocarcinomas and squamous cell carcinomas [2]. Approximately 75% of BCs are classified as pure urothelial carcinomas, and the remaining 25% are urothelial and nonurothelial tissues [3]. BC can be classified into either muscle invasive BC (MIBC) or non-muscle invasive BC (NMIBC). The proportion of NMIBC is 70% in the first diagnosed, based on the depth of invasion of the bladder wall. For MIBC with nonmetastatic disease, the 5-year overall survival rate is 36%-48%, while the 5-year overall survival rate in MIBC with distant metastasis and regional metastasis is 5% and 36%, respectively [4]. Patients with BC have variable prognoses due to different clinical characteristics and pathological classifications. Current standard therapy for NMIBC is transurethral resection of the bladder tumour (TURBT), and subsequently, the implementation of various chemotherapies. Radical cystectomy depends on whether BC metastasizes to MIBC, and then chemotherapy, immunotherapy, and targeted therapy are performed [4]. Despite many advances in BC diagnosis and therapy, a high rate of metastasis, drug resistance and tumour recurrence, resulting from remarkable genomic instability and high intratumor heterogeneity remain to be overcome [5, 6]. A proper orthotopic animal model that is reproducible, reliable, and simple plays an important role in studying the mechanism of BC.

A murine BC model has many controllable variables under laboratory conditions, offering many experimental parameters [7]. Previous studies indicate that the ideal BC animal model is described as follows: (1) it occurs in the bladder; (2) it allows tumours to originate from urothelial cell carcinoma; (3) it grows via an immunocompetent host; and (4) it is operationally easy to reproduce [8].

In this study, we reported an improved orthotopic BC model established with microsyringes in mice. Our aim was to provide a stable, reproducible, convenient method for constructing a murine BC model in male mice. Meanwhile, the model can offer a useful tool for studying the growth, invasion, metastasis, drug resistance and therapy of tumours.

## Methods

### Chemicals and equipment

**Fig. 1:**
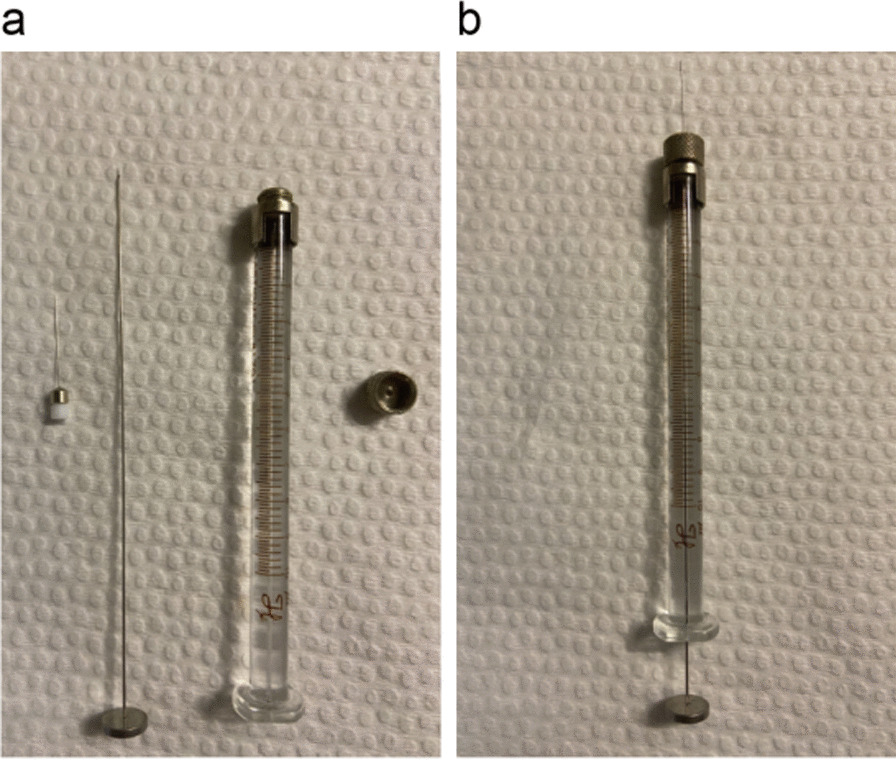
Microsyringes in scattered (**a**) and assembled (**b**)

Dulbecco’s modified Eagle’s medium (DMEM), phosphate buffered saline (PBS), foetal bovine serum (FBS), trypsin and penicillin–streptomycin solution were obtained from *Gibco* (*Gibco, USA*). Depilatory cream was purchased from *Veet (Veet, USA)*. Pentobarbital sodium was provided by Professor Chen She from Fudan University. Microsyringes were purchased from *Hamilton* (*Hamilton, USA*) (Fig. 1). A multi-mode ultrasound/photoacoustic imaging system for small animals was provided by the instrumental analysis centre of Shanghai Jiao Tong University.

### Cell strain

The murine BC cell line MB49 was purchased from Shanghai Institutes for Biological Science, CAS. MB49 cells were derived from C57BL/6 mice via exposure of murine bladder urothelial cells to 7,12-dimethylbenz[a]anthracene (DMBA) for 24 h followed by long-term culture [9]. The cells were cultured in DMEM supplemented with 100 U/mL penicillin–streptomycin solution and 10% FBS at 5% CO_2_ and 37 ℃. The cells were digested using trypsin, washed with PBS, and then resuspended in PBS at 5 × 10^6^/mL. Finally, the prepared cells were placed on ice.

### Orthotopic transplantation operation

**Fig. 2:**
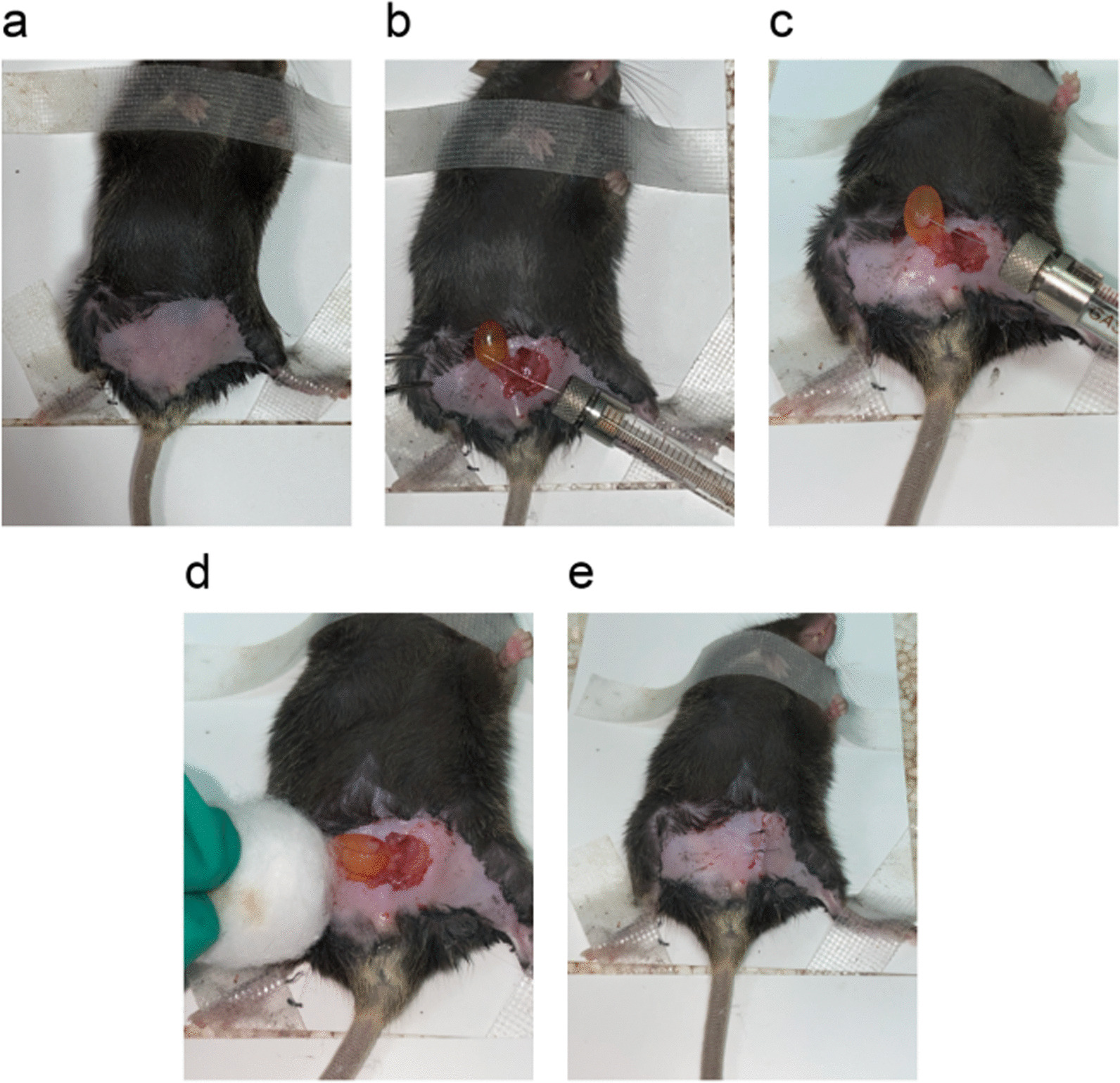
The operation procedures of orthotopic transplantation. **a** The lower abdomen area was depilated and disinfected. **b** The bladder was dissected and identify vascular-enriched area. **c** Gentle inject cells. **d** Observation after injection. **e** Close the lower abdomen and disinfect

Mice were anaesthetized with pentobarbital sodium solution at a dose of 0.14 mL/10 g body weight. Subsequently, the lower abdomen of the mouse was gently massaged to expel the remaining urine, depilated, and disinfected. A 10 µL microsyringe was utilized to withdraw 10 µL of the suspension after it was drawn up and down through the 1 mL pipettor. The approximate abdominal area of the bladder was then identified, and the cavity was opened. The bladder was found and pulled out of the body so that the needle of the microsyringe was inserted into the vascular-enriched area of the bladder wall. Cells were gently injected into the bladder wall, and a white bump was observed. The wound was sutured with 6-0 nylon sutures layer by layer. The main procedures are shown in Fig. 2.

### Animals

Our present experiments were approved by the local ethics committee and the Shanghai Medical Experimental Animal Care Commission. To establish the improved orthotopic model, six-week-old male C57BL/6 mice were purchased from Beijing Vital River Laboratory Animal Technology Co., Ltd. Mice were fed in a pathogen-free animal facility under standard conditions. All animals were randomly assigned to either the experimental or control group. All animal operations were performed according to the criteria and principles in the “Guide for the Care and Use of Laboratory Animals” published by the National Academy of Sciences and prepared by the National Institutes of Health (NIH Publication 86-23 revised 1985).

### HE staining

Specimens collected after BC included bilateral ureters, liver, kidneys, heart, spleen and lungs. They were washed with PBS, fixed in formalin for 24 h, dehydrated with 70%, 80%, 90% ethanol and absolute alcohol for 5 h, and then vitrified with xylene I and xylene II for 20 min. The specimens were embedded and sliced after immersing in paraffin I and II for 1 h. Staining was completed as follows: haematoxylin staining for 10 min, hydrochloric acid alcohol solution for 20 s destaining, eosin staining for 2 min and 90% ethanol for 40 s destaining. Finally, the section was mounted with neutral balsam and observed and photographed under a microscope [10].

### Statistical analysis

If data did not conform to a normal distribution, the Mann–Whitney test was used. Statistical analysis between groups for significance was completed using Student’s *t*-test. The results were analysed using GraphPad 8.0 software. The results of the analysis are presented as the mean value ± SD. The level of significance in the statistics was set at *P < 0.05; ** *P* < 0.01; ***P < 0.001.

## Results

### The evaluation of tumour progression

**Fig. 3:**
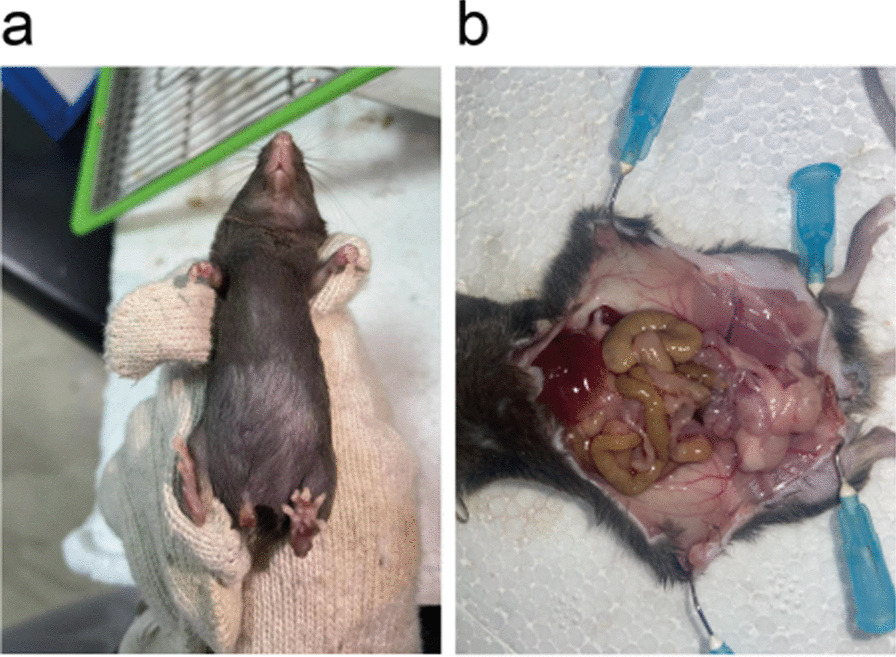
Exhibition of murine hematuresis (**a**) and abdominal organs (**b**)

In our study, a total of 30 C57BL/6 mice in the modelling group were injected with a 10 µL suspension in the bladder wall. Mice in the control group underwent sham surgery under the same conditions. Murine haematuresis was observed on the 17th day, as shown in Fig. 3a. The whole observation was suspended on the 21st day. Then, murine abdominal organs were exposed, and no typical peritoneal metastasis was found (Fig. 3b).

**Fig. 4:**
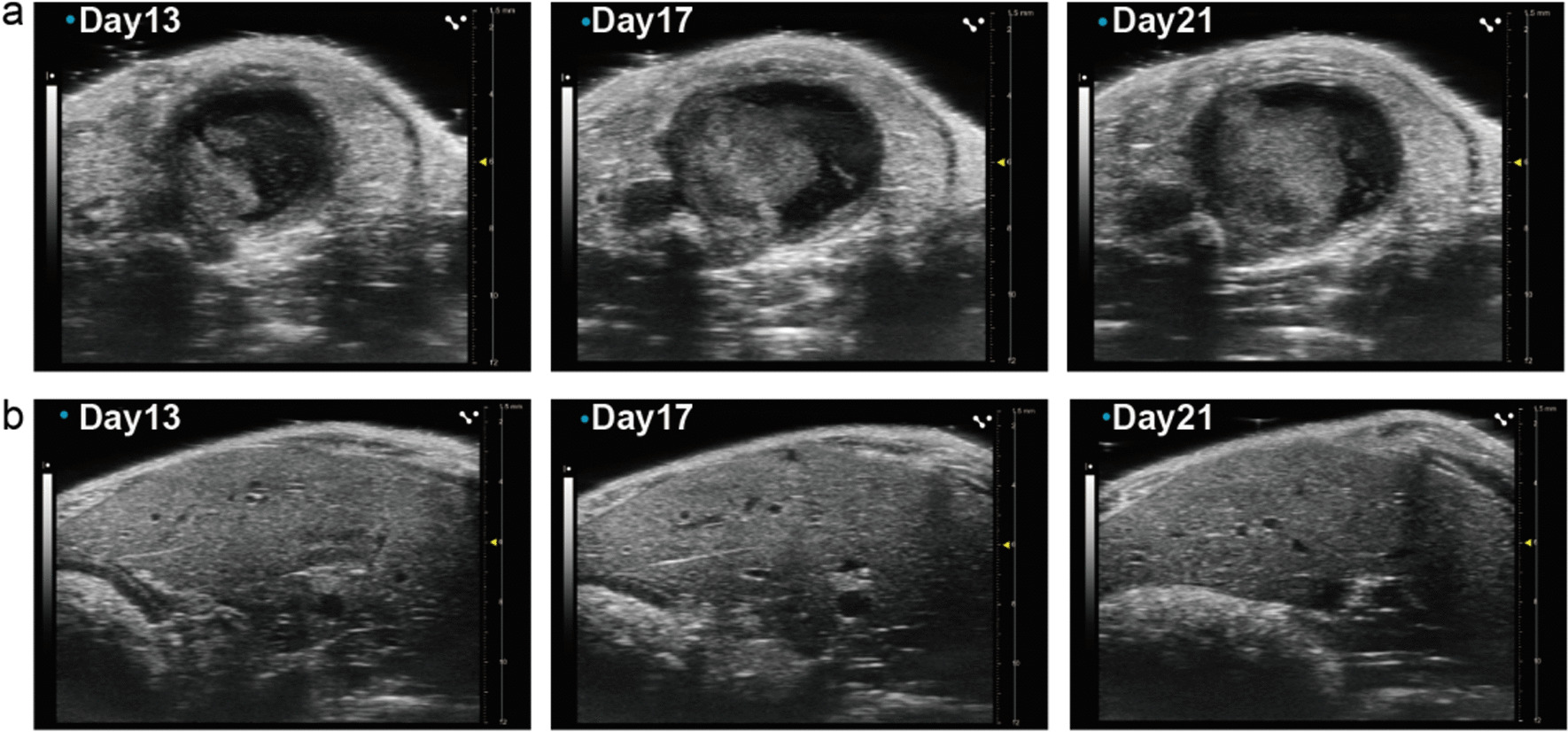
TUS of a mouse bladder tumor and liver. **a** The growth of bladder tumor can be observed and measured longitudinally via TUS. **b** The liver can be monitored by TUS during the observation of bladder tumor

Furthermore, with the help of transabdominal ultrasonography (TUS), the growth process of bladder tumours was detected every 3 days from the first day to the last day in both groups. Meanwhile, the liver was monitored during the observation of bladder tumours due to the excellent performance of TUS. The visible tumours were detected on the 13th day, and the larger tumours were monitored in later observations on the 17th and 21st days (Fig. 4a). No significant difference was observed from the imaging data, as shown in Fig. 4b.

### The analysis of survival time

**Fig. 5:**
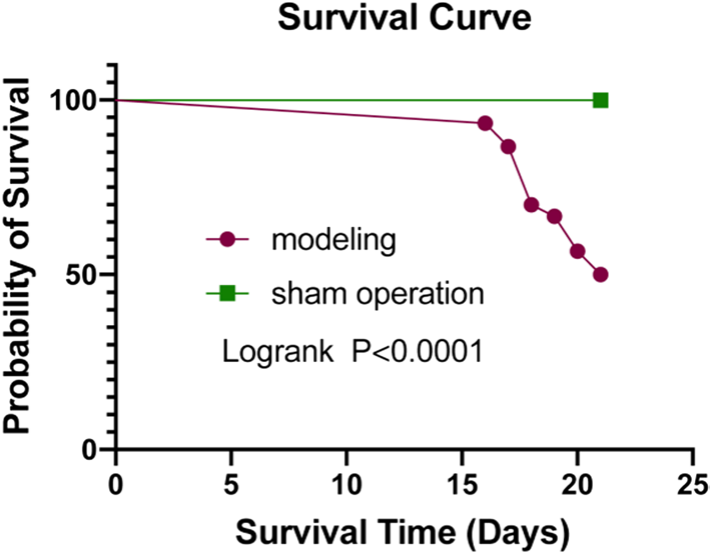
Survival time of the modelling and the sham operation

Mice began to die on the 16th day in the modelling group following cell inoculation. Half of the mice in both groups died, which denoted the end of the experiment. The remaining mice were sacrificed after the experiment was completed. There were 29 C57BL/6 mice with tumours in the modelling group, while tumours did not occur in mice that received a sham operation. The tumour incidence was 96.67%. The average survival time in the modelling mice was (19.73 ± 1.69 d) (Table 1). The average survival time in the modelling mice was distinctly lower than that in the control group (*P* < 0.0001), which is displayed in Fig. 5.

**Table 1:** Tumor incidence, average survival time, average bladder wet weight, average mouse weight and tumor volume

Groups	Number of mice	Number of tumor developed mice	Tumor incidence (%)	Average survival time (days)	Average bladder wet weight (g)	Average mouse weight (g)	Average tumor volume (mm^3^)
Modelling	30	29	96.67	19.73 ± 1.69	0.0385 ± 0.0048	27.40 ± 0.82	2.58 ± 2.50
Control	30	0	0	> 21	0.0345 ± 0.0056	28.06 ± 1.07	–

### Metastasis in the modelling group

**Fig. 6:**
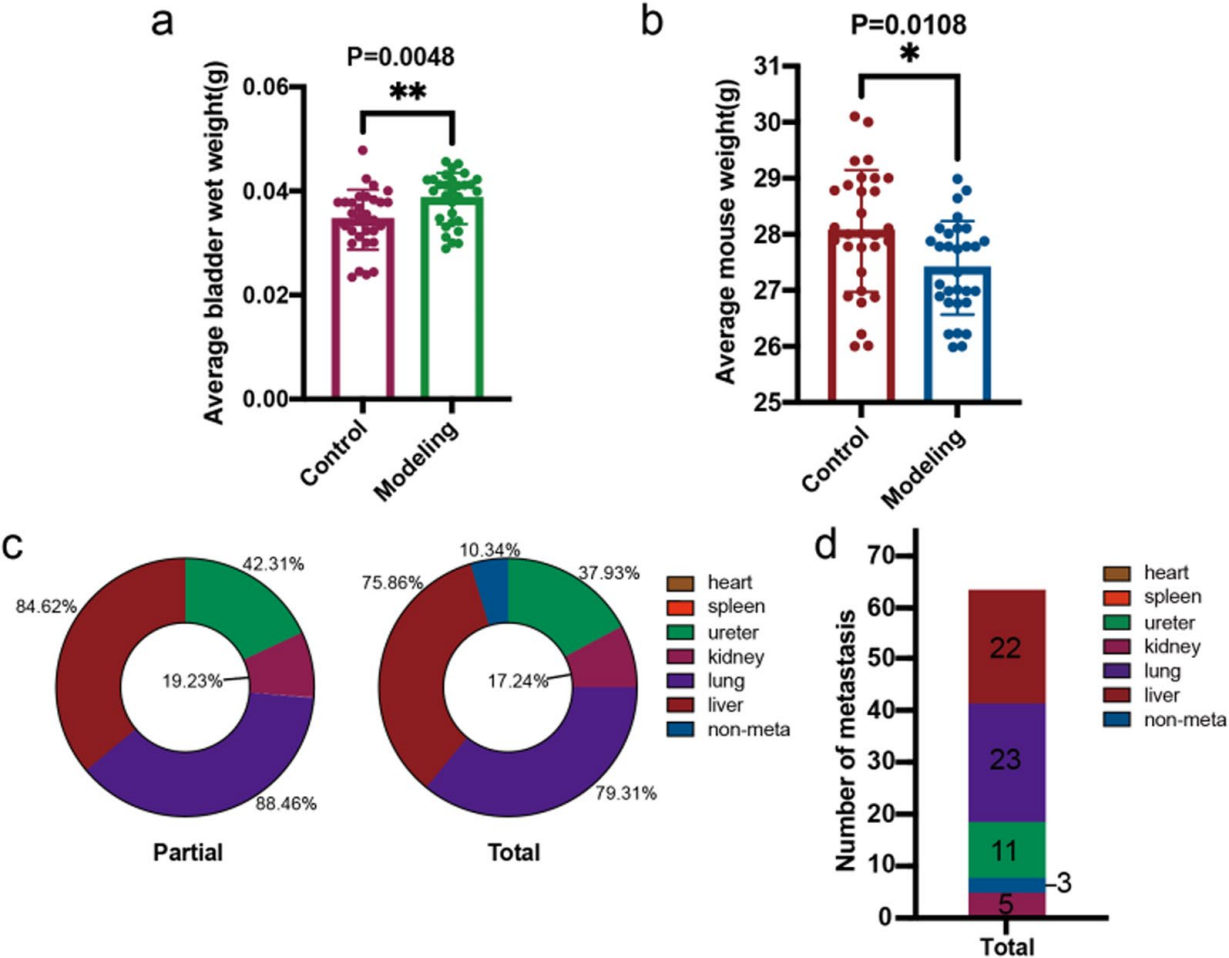
Comparison of average bladder wet weight (**a**) and average mouse weight (**b**) and metastasis analysis (**c**, **d**)

As the picture shows (Fig. 2c), a white bump was formed by resuspended cells. Meanwhile, vascular wall was intact. And the needle did not break through vascellum so that the injected cells did not go directly into the systemic circulatory system. With the help of invasive characteristic of tumour cells, tumour progression and metastasis happened in that context. There were 26 C57BL/6 mice that were observed to have metastasis (Fig. 6d), which was found in the lungs (23/26), liver (22/26), ureter (11/26) and kidneys (5/26) (Fig. 6d). The metastasis rate of all mice was 89.66%. A total of 4 organs (lungs, liver, ureter, kidneys) were observed to have metastasis (Fig. 8b, c, f, g), while the spleen and heart were not found to have metastasis (Fig. 8a, d). The metastatic rate of the lungs, liver, ureter and kidneys was calculated as 88.46%, 84.62%, 42.31% and 19.23% of the metastatic mice in the modelling group, respectively (Fig. 6c), and the proportion of metastasis in the lungs, liver, ureter, and kidneys in nonmetastatic mice accounted for 79.31%, 75.86%, 37.93%, 17.24% and 10.34% in the modelling group, respectively (Fig. 6c).

### The assessment of pathology

**Fig. 7:**
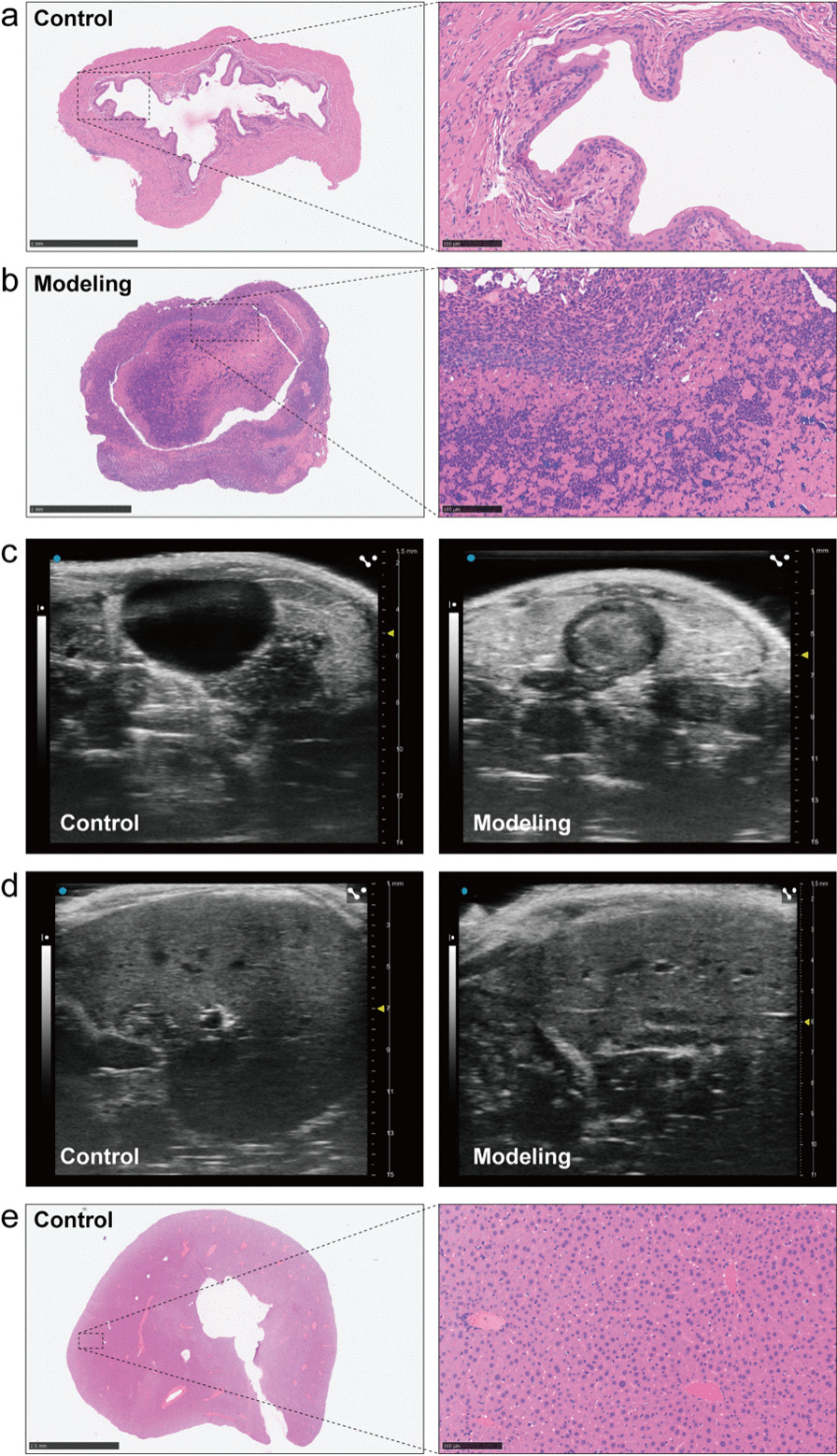
Pathological images and TUS of mice bladder and liver in the control and modelling group. **a**, **b** Pathological images (HE staining) of bladder tumor in the control group (sham operation) and the modelling group. **c**, **d** Bladder and liver of TUS of bladder tumor in the control group (sham operation) and the modelling group. **e** Pathological images (HE staining) of liver in the control group

An intact, smooth bladder mucosa was observed via HE staining and TUS in the sham operation group (Fig. 7a, c). Bladder tumours in the modelling group were confirmed via HE staining (Fig. 7b), while mice that underwent the sham operation had normal bladders (Fig. 7a). The TUS image showed the same result (Fig. 7c). There was no distinct difference between the two groups (Fig. 7d), and the liver in the control group was not found to have cancerous nodes via HE staining (Fig. 7e). No metastases were detected by the same method in other abdominal organs. Compared with the control group, the modelling mice had serous cachexy and visible haematuresis. The mouse weight data of both groups were analysed, which indicated that the weights in the control group were significantly higher those in the modelling group (*P* = 0.0108) (Fig. 6b). The average weight in the modelling mice was 27.40 ± 0.82 g; on the other hand, the average weight in the control mice was 28.06 ± 1.07 g. Subsequently, each bladder in the two groups was weighed, and the results showed that the average wet bladder weight in the modelling mice (0.0385 ± 0.0048 g) was significantly higher than that in the control mice (0.0345 ± 0.0056 g) (*P* = 0.0048) (Fig. 6a, Table 1). The average tumour volume was calculated to be 2.58 ± 2.50 mm^3^ (Table 1).

**Fig. 8:**
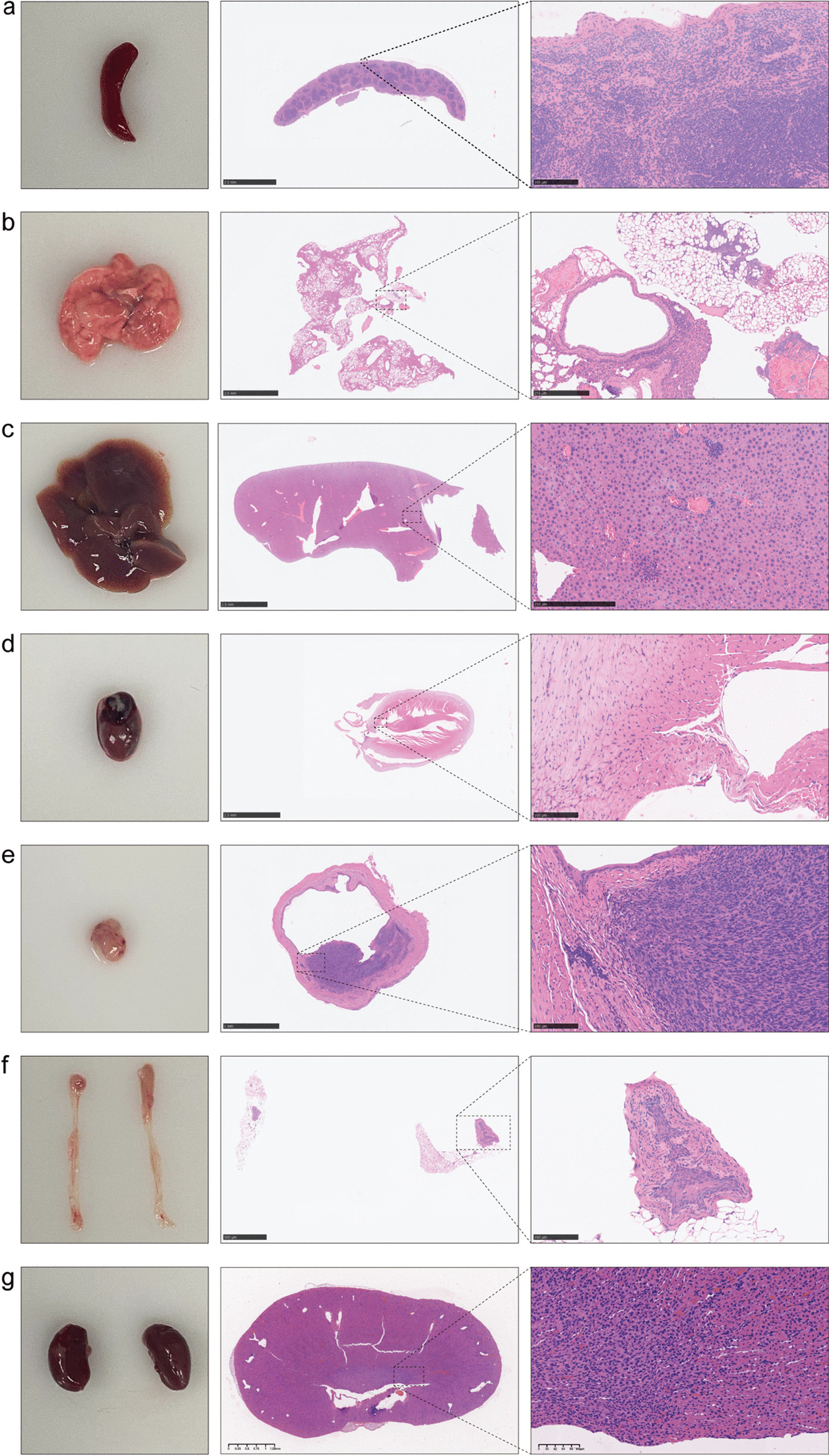
Pathological images of spleen (**a**), lung (**b**), liver (**c**), heart (**d**), bladder (**e**), ureter (**f**), kidney (**g**)

Tumour cells infiltrated deeply into the muscular layer, which shows typical cellular atypia (Fig. 8e). Interestingly, tumour morphogenesis of cellular shape and disordered arrangement indicate the loss of cell polarity (Figs. 5b, 8e). Some tumour slices were observed to have normal urothelium. Furthermore, perimeter cells of low-grade bladder tumours had the characteristics of a slightly rounded and relaxed overall surface with a more regular arrangement (Fig. 8e). The high-grade internal tumour cells have a twisted and crowded shape with a nucleus that has an irregular appearance. A large number of cells in the central part of the tumour exhibited necrosis due to the lack of blood supply (Fig. 5b). All pathological results from tumour-bearing mice were confirmed to be MIBC by two attending urologists.

## Discussion

Given the global incidence and mortality of BC, research on BC is very important. The orthotopic murine transplantable model of BC is a practical model which takes into account the clinical pathological analogy to humans [11]. Murine orthotopic BC models can be divided into two groups: (1) nonautochthonous models and (2) autochthonous models. Nonautochthonous models often utilize orthotopic engraftment in which murine syngeneic or human BC cells adhere to the bladder wall of normal or immunodeficient recipient hosts. Moreover, BC cells can also be injected into the wall of the bladder, which plays the same role as orthotopic engraftment, allowing tumours to grow in the bladder [12–17]. Autochthonous models consist of orthotopic carcinogen-based models and transgenic or genetically engineered mice (GEMs). The first orthotopic BC model was induced with carcinogens in rats, and the most common carcinogen used to induce BC in mice is N-butyl-N-[4-hydroxybutyl] nitrosamine (BBN) [18–20]. Transgenic mice or GEM orthotopic models are generated to either carry cloned oncogenes or lack tumour suppressing genes, including the Cre-loxP system and the injection of adenovirus-Cre in the lumen of the bladder [21–24]. As demonstrated above, there are four most frequently used methods to construct orthotopic murine models of BC. First, a tumour cell suspension is instilled into the bladder via catheter or laparotomy [25, 26], which utilizes N-methyl-N-nitrosourea or a mixture of chlorhydric acid and potassium hydroxide [8, 27, 28]. This method does not lead to urinary stones or traumatic ulceration, and its rate of tumour implantation ranges from 28 to 100%. The tumour occurrence is multifocal and unpredictable in localization. Moreover, operations are invisible because the process occurs inside the bladder so that the success rate is unreliable. Second, weak acid, protease or other chemicals are injected into the bladder mucosa for corrosion before tumour cells are injected [29–31]. This method requires complicated experimental conditions, such as corrosion time, and the tumour-bearing rate is unstable. The third method utilizes mechanical tools to injure the bladder mucosa before tumour cells are injected via inserting a stylet or other tools [32]. All operations happen inside the bladder so that the process is uncontrolled and the success rate is uncertain. The fourth method resembles our operation, in that it uses a regular injector to inoculate mice. However, the volume of the cell suspension cannot be controlled. Abdominal metastasis happens unpredictably, resulting in the obstruction of experimental conclusions or the addition of interference factors [33–37].

Transabdominal operation is one of the most controlled methods of constructing a murine orthotopic model in which the procedures are visible. And different pathological types have an impact on clinical outcomes, such as non-urothelial variant histology (VH) bladder cancer (BC) and urothelial carcinoma of the urinary bladder (UCUB) [38]. Despite the technical difficulty of orthotopic tumour cell transplantation, this method improves the tumour-bearing rate and avoids pathological variants. Intravesicular instillation of tumour cell suspensions via catheters or stylets is currently one of the most frequent methods used to establish orthotopic murine models of BC with or without injury. Interestingly, female mice can only be used because of anatomical features [39, 40]. Our hormone research results indicate that the level of oestrogen has a significant impact on the progression of BC (unpublished data). Although the metastatic organs did not have typical cancerous nodes, tiny cancerous nodes were found under the microscope. Previous studies of the association between oestrogen and BC have demonstrated some mechanisms [41, 42]. Moreover, the epidemiology of BC and related mechanical studies have confirmed the phenomenon and mechanism [43–46]. Our previous study found that the loss of cell polarity has an effect on tumour metastasis in BC [47], which is consistent with our present results that show that the optimized orthotopic model has a high metastatic rate. Therefore, the orthotopic BC model in our study is theoretically practical to figure out the clinical pathological process in humans. By analogy, the optimized model can be utilized in immunodeficient mice and even rats, and can be constructed under the specific conditions of human BC cells.

## Conclusion

Herein, we describe a new murine orthotopic BC model that is reproducible. The model has had a major impact on helping us improve our knowledge of the mechanisms and pathogenesis of BC at the proper anatomical site. In conclusion, we succeeded to establish an improved murine orthotopic BC model derived from MB49 cells using a microsyringe. The model offers a reproducible, rapid, convenient tool to carefully study the events in multistep cascades of tumour initiation, progression, therapy and so on.

## Data Availability

The datasets used during the current study available from the corresponding author on request.

## Abbreviations

BC: Bladder cancer
TUS: Transabdominal ultrasonography
GEM: Genetically engineered mice
BBN: N-butyl-N-(4-hydroxybutyl) nitrosamine
